# Clear cell carcinoma of the ovary: multi-slice computed tomography findings

**DOI:** 10.1186/s12957-015-0546-1

**Published:** 2015-04-01

**Authors:** Xubin Li, Zhaoxiang Ye

**Affiliations:** Department of Radiology, Tianjin Medical University Cancer Institute and Hospital, National Clinical Research Center for Cancer, Key Laboratory of Cancer Prevention and Therapy, Huan-hu-xi Road, Hexi District 300060 Tianjin, China

**Keywords:** Ovary, Neoplasms, Clear cell carcinoma, X-ray, Computed tomography

## Abstract

**Background:**

The aim of the study was to describe the multi-slice computed tomography (MSCT) features of clear cell carcinoma (CCC) of the ovary.

**Methods:**

Twenty patients with histology-confirmed CCC of the ovary were retrospectively reviewed. All patients underwent preoperative plain and contrast-enhanced MSCT examinations. Imaging studies were evaluated for the following: (a) location; (b) maximal transverse diameter; (c) shape (round, oval, lobular, or irregular); (d) margin (well defined or ill defined); (e) solid, solid with cystic regions, or cystic; (f) attenuation of the cystic portion; (g) enhancement pattern of the solid portions of the tumor; and (h) the secondary manifestations.

**Results:**

The mean age of the patients was 63 years (range, 44 to 77 years). Tumors were unilateral in 19 patients and bilateral on 1 patient. The maximal transverse diameter of the tumors was relatively larger with a mean diameter of 105.7 mm (range, 45.5 to 260.6 mm). CCCs demonstrated cystic masses with solid regions in 20 lesions. Most lesions were ovoid (15/21), unilocular (16/21), and well-defined (17/21). The CT value of cystic or necrotic portion ranged from 12 to 28 HU (average, 18 HU) on plain image. The solid protrusions of the cystic masses were both few and round with obviously heterogeneous enhancements after contrast.

**Conclusions:**

The ovarian CCCs typically present as a large, well-defined, unilocular cystic mass with solid protrusion and relatively high attenuated cystic or necrotic portions. The solid protrusions are usually both round and few in number with obviously heterogeneous enhancement.

## Background

Clear cell carcinoma (CCC) of the ovary, a rare histological subtype of ovarian epithelial carcinoma, is now recognized as a unique disease with specific developmental origin activated molecular pathways and clinical features [1]. CCCs are biologically aggressive neoplasms and remarkably resistant to conventional platinum-based chemotherapy. However, early-stage CCCs have an excellent prognosis and may not require any adjuvant therapy [2]. Therefore, preoperative accurate diagnosis of CCC may be important for its optimal therapeutic strategy.

Because of CCC’s low prevalence, previous reports on imaging findings of ovarian CCCs are few and most reports focused on magnetic resonance imaging (MRI) [3,4] or contained small case series [5]. The development of multi-slice computed tomography (MSCT) technology and helical scanning techniques has revolutionized the use of MSCT for primary diagnostic evaluation of ovarian lesions. The distinct MSCT features of ovarian CCC have not been well described. Therefore, the objective of this study was to investigate the MSCT characteristics of ovarian CCC in order to enhance the awareness of this uncommon entity among both clinicians and radiologists.

## Methods

### Patients

This retrospective study was approved by the institutional review boards of our hospital (Tianjin Medical University Cancer Institute and Hospital, Tianjin, China), wherein the requirements for informed consent were waived. Twenty patients with histology-confirmed ovarian CCC between October 2011 and March 2014 were enrolled in this study. All patients underwent plain and contrast MSCT examinations and then surgical removal of CCCs. None of the patients underwent biopsy before MSCT scanning. Their clinical presentations and histological diagnosis were extracted from the medical records.

### MSCT protocols

MSCT scanning was performed by using a LightSpeed VCT (GE Healthcare, Milwaukee, WI, USA) through the whole pelvis. The images were acquired with a contiguous section thickness of 1.25 mm, a field of view of 400 × 400 mm, a peak voltage of 120 kVp, a tube current of 200 mA, and a matrix of 512 × 512. For contrast-enhanced imaging, a bolus intravenous dose of 80 ml of nonionic iodinated contrast agent (Ultravist 300; Schering, Berlin, Germany) was administered using a power injector (Multilevel CT; Medrad, Pittsburgh, PA, USA), through an 18-gauge intravenous catheter placed in the antecubital vein at a rate of 2.5 ml/s. After the contrast material injection, 20 ml of normal saline was administered immediately. The scanning was initiated 60 s after the onset of contrast injection. The axial images were reconstructed in both sagittal and coronal planes with a section thickness of 3 mm.

### Image analysis

Two radiologists in gynecologic imaging evaluated all images retrospectively for a consensus opinion. The following morphologic features were carefully evaluated: (a) location; (b) maximal transverse diameters; (c) shape (round, oval, lobular, or irregular); (d) margin (well defined or ill defined); (e) solid, solid with cystic regions, or cystic; (f) attenuation of the cystic portion (lowest value measured in the cystic or necrotic portion of the mass without enhancement); (g) enhancement pattern of the solid portions of the tumor; and (h) the secondary manifestations (the involvement of local lymph nodes, adjacent organs, and ascites). The enhancement patterns of the solid portions were divided into homogeneous and heterogeneous enhancements.

## Results

The patients enrolled in the present study ranged in age from 44 to 77 years with a mean age of 63 years at diagnosis. Fourteen patients were postmenopausal. Seventeen patients presented with a palpable mass without tenderness, while three patients had slight abdominal pain. Twenty-one lesions of CCC of the ovary were finally found. CCCs were observed in bilateral ovaries in only one case. Eleven were on the right and ten were on the left. In our series, due to our limited awareness of this uncommon tumor, 7 cases were diagnosed as ovarian cystadenocarcinoma including bilateral cystadenocarcinomas in 1 case, and 13 cases were diagnosed as ovarian cystadenoma before surgery. The maximal transverse diameter of the tumors ranged from 45.5 to 260.6 mm with a mean diameter of 105.7 mm.

**Figure 1:**
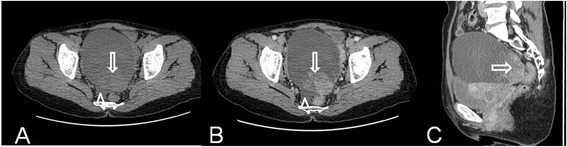
**Ovarian clear cell carcinoma in a 49-year-old woman.** Axial precontrast MSCT image **(A)** shows a mostly cystic, unilocular, and oval-shaped mass with smooth margin and irregular solid protrusion (arrow). Axial **(B)** and sagittal **(C)** contrast-enhanced CT images show obviously heterogeneous enhancement of the solid component in the tumor (arrow). Note the minimal fluids (arrowhead).

**Figure 2:**
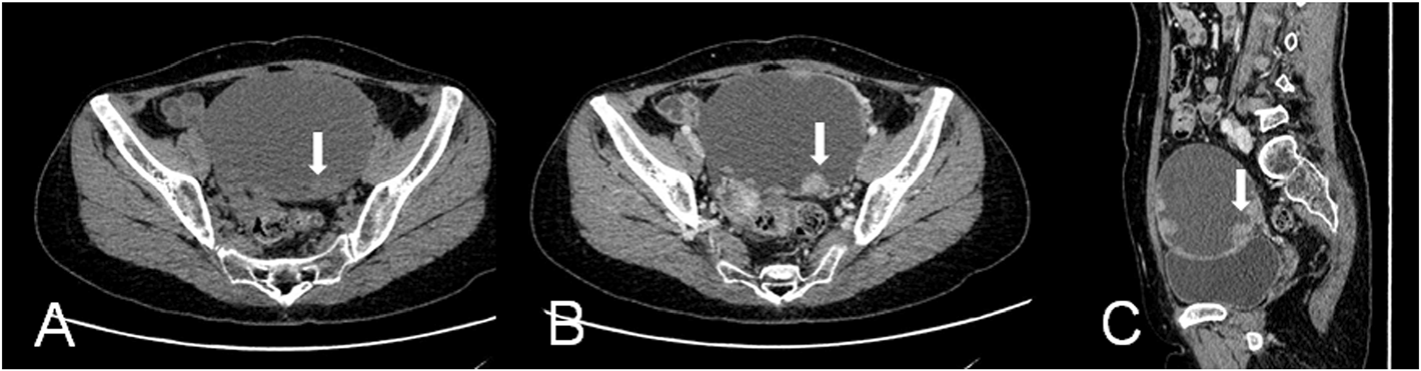
**Ovarian clear cell carcinoma in a 47-year-old woman.** Axial precontrast MSCT image **(A)** shows a mostly cystic, unilocular, and oval-shaped mass with smooth margin and round solid protrusion (arrow). Axial **(B)** and sagittal **(C)** contrast-enhanced CT images show obviously heterogeneous enhancement of the solid component in the tumor (arrow). The solid protrusions are both round and few in number.

MSCT imaging of CCCs demonstrated cystic masses with solid regions in 20 and solid masses in 1. The shape of the CCCs was found to be round (*n* = 1), ovoid (*n* = 15), lobular (*n* = 1), or irregular (*n* = 4), respectively. The margin was ill-defined in 4 tumors and well-defined in the remaining tumors. The 20 cystic masses were multilocular in 4 masses and unilocular in 16 lesions (Figures 1 and 2). The attenuation of cystic or necrotic portion ranged from 12 to 28 HU (average, 18 HU) on plain MSCT images. The solid protrusions of the cystic masses were few in number, round in 15 lesions (Figure 2), and irregular in 5 lesions (Figure 1). The solid components of the CCCs showed obviously heterogeneous enhancements after contrast injection (Figures 1 and 2). The involvements of local lymph nodes in 2 patients and adjacent organs in 4 patients were identified, which were confirmed by pathology. Massive or moderate malignant ascites were found in 7 patients, and minimal benign ascites were found in 2 patients (Figure 1).

## Discussion

CCCs of the ovary are now widely accepted as a unique disease with specific developmental origin activated molecular pathways and clinical features. It is a rare subtype of ovarian carcinomas and accounts for approximately 5% of ovarian carcinomas. The mean age of patients presenting with CCC is approximately 50 years [3]. In the present study, CCC mainly affected women in the postmenopausal years (14/20) with a mean age of 63 years and no distinctive clinical symptoms. The tumors were unilateral in 19 patients (19/20), which was consistent with the previous reported literature [6]. According to the literature, distant organ and lymph node involvements are frequent in patients with recurrent CCC [7]. However, contrast to the literature, the involvements of local lymph nodes in 2 patients and adjacent organs in 4 patients with primary tumors at diagnosis in our cases were identified.

It is clinically important to differentiate clear cell cancer from other ovarian cancers, because the prognosis of CCC appears to be better than that of other ovarian cancers after resection at stage I [8] and MSCT has become a useful tool to allow local and distant staging of ovarian cancer in a single examination with the ability of performing a quick one-stop examination of the whole abdomen and pelvic. Based on MSCT appearances in our series, most ovarian CCCs (20/21) showed larger cystic masses with solid regions. There were 16 lesions demonstrating a unilocular cystic mass. Most tumors (17/21) had a well-defined margin. The attenuation of the cystic portion of the mass was relatively high (average, 18 HU). For the cystic portion with attenuation higher than 20 HU in 4 lesions, the contents were necrosis or hemorrhage, and for the cystic portion with attenuation lower than 20 HU, the content was serous fluid, which was confirmed by pathology and consistent with the previous report [5]. This is regarded as a differential point from the other common epithelial origin tumor such as cystadenocarcinoma, because the cystic content of cystadenocarcinoma usually has attenuation similar to that of water. For the solid protrusions of the cystic masses, they were relatively small in number, round in 15 lesions and irregular in 5 lesions. These findings coincide with the previous report [3]. After contrast, the solid components of the CCCs showed obviously heterogeneous enhancements, which suggested their hypervascular characteristics. It indicates, but is not specific for, malignant tumors.

Large peritoneal fluid pockets and peritoneal enhancements are predictive for malignancy [9]. In our series, massive or moderate malignant ascites were found in 7 patients, and minimal benign ascites were found in 2 patients. Peritoneal lesions were not found by MSCT before surgery, which was confirmed by pathology after surgery.

There were some limitations to our study. The main limitation was that the number of ovarian CCCs that we assessed was relatively small for the analysis of distinct MSCT features. Secondly, comparisons with other ovarian neoplasms were not established in our study because of the low incidence of ovarian CCC. Finally, long-term follow-up with either MSCT or MRI scans were not well performed for the patients, and thus, the exact recurrence and metastasis of ovarian CCC could not be accurately documented.

## Conclusions

Ovarian CCCs typically present as a well-defined, unilocular cystic mass with solid protrusion and relatively high attenuated cystic or necrotic portions. The solid protrusions are usually both round and few in number with obviously heterogeneous enhancements. Although these findings most likely indicate a CCC of the ovary, the differential diagnosis should include a serous tumor with low malignant potential or a serous cystadenocarcinoma. Further research with larger cases is clearly needed for this rare type of ovarian carcinoma.

## Consent

Written informed consent was obtained from the patients for publication of this paper and any accompanying images. A copy of the written consent is available for review by the Editor in Chief of this journal.

## Abbreviations

CCC: clear cell carcinoma
MSCT: multi-slice computed tomography
